# Reports on the Medical College of Ohio

**Published:** 1838

**Authors:** 


### Art. II.—Proceedings in the Legislature of Ohio on the Medical College of Ohio.

We give two Reports, by a Standing Committee of our Legislature on this Institution. The Committee is composed of five members, two of whom, we are informed, were in favor of one report, and two of the other—one taking part in neither. The first, however, is called the majority report, because, as we presume, it was made by the Chairman of the Committee. At this date, March 17th, when we are obliged to send the reports to our printer, we have not heard what action the General Assembly has had upon them. We present them to our readers, without note or comment.

Mr. Bronson, from the Committee on Medical Colleges and Medical Societies, made the following Report:

The committee on Medical Societies to whom was referred the report of the Trustees of the Ohio Medical College, with instructions to inquire into "the causes if any, which have retarted its progress or limited its usefulness as a public institution," have had the same under consideration, and now Report:

The resolution of instruction does not limit the investigation of the committee to any particular period of time, and it has been deemed proper to embrace a period somewhat remote. In doing so, however, the committee has thought proper to refer to information furnished the Legislature in the winter of 1833–4, as being the best and most conclusive within their reach. During the preceding session of the Legislature, various charges had been seriously preferred, and many *calumnies*, as your committee believe, circulated against the Ohio Medical College, its faculty and board of Trustees. With a view to ascertain the correctness of these charges, a select committee was appointed, clothed with power to send for persons and papers, and after a full, tedious and patient examination of witnesses on the various charges made, as stated above, both on the part of the prosecutor and the College, the committee paesented its report, accompanied with a large mass of written evidence.

This report, together with the testimony accompanying it was made out during the summer of 1833, and transmitted to the Legislature the following winter, when it was referred to an appropriate committee.—The large mass of testimony was carefully examined and arranged, and presented to the Senate in a report from the committee, through their chairman, Judge Hitchcock. To those who feel an interest in this matter, either personally, professionally, or as members of the Legislature, the committee would recommend the above report as containing a large amount of interesting evidence, and conclusive reasoning in a small compass. The report concludes in this language: "Taking into consideration all the circumstances, the committee believe that no further Legislative action is necessary;" and after asking to be discharged from the further consideration of the subject, a vote was taken upon agreeing to the report, and carried with but one dissenting voice.—From an examination of this report and the decision upon it, the committee cannot but conclude that the injurious charges made against the Ohio Medical College, up to the period when the above report was presented to the Senate, were altogether groundless.

The condition of the school during the last three years, cannot be ascertained satisfactorily, except by the means of the annual reports of the board of Trustees. Their last annual report contains a candid and satisfactory statement of the causes, which led to the vacation of several of the chairs in the Ohio Medical College, during the summer of 1837. The school had obtained the confidence of a widely extended profession; the number of Students had largely augmented, with the most flattering prospect of a continued increase, when, from motives, into which it is not proper to inquire, large sums were offered for the services of some of the Professors, and they were withdrawn to chairs in other rival institutions. The condition into which the Ohio College was thrown by these changes, could not but for a time operate to the injury of the institution. Changes in the Faculty or in the Trustees of such institutions, unless morally or intellectually disqualified, have always had a pernicious influence. In the one case, all aspirants cannot be satisfied, and it is unnecessary to say in what direction disappointed ambition, is most likely to direct its influence. In the other case, there is apt to be an angry and unprofitable struggle, to elect such men as would be most likely to advance particular interests.

Frequent legislation respecting the condition of a public institution, especially when it directs an inquiry similar to th one contained in the resolution upon which this report is founded, is always more or less injurious. It does not require the direct charge of guilt, to fix suspicion either upon the character of an individual, or upon that of a public institution. Many will draw an unfavorable inference from a mere hint that an investigation is necessary, while it will afford assistance to those who may be cherishing designs toward either.

Medical Colleges can only be successfully conducted and sustained in large cities, where students can have an opportunity of witnessing hospital practice and surgical operations. Anatomy cannot be correctly taught, without subjects; chemistry, without a laboratory; surgery, without living subjects, nor the theory and practice of medicine, without an extensive hospital. Cincinnati, your committee believe, combines all these advantages, and to remove the institution from its present location to one in which few, if any, of the above advantages could be enjoyed, would be little less than destruction; and to inspire a belief that such a removal is contemplated, by a mere agitation of the question, is not likely to establish confidence in the stability of the institution.

Connected with the Ohio Medieal College is a hospital, in which are constantly found from one to two hundred patients. These patients receive the professional attendance of the Faculty of the Medical College, who are thereby enabled to present to students, cases of a vast number, if not all, the diseases which they may be called upon to treat; and many important surgical operations have recently been performed by Professor Murphy, before the present class. Both the Medical and Surgical departments are at present so well conducted as to induce many who are possessed of means to defray their own expenses, to enter the hospital and solicit professional attendance in that place, in preference to seeking aid from other sources. These, added to the sick who are provided for by the county of Hamilton and city of Cincinnati, and the sick boatmen who are supported by the General Government, will always furnish a numerous and increasing supply of patients.

Unfavorable as were the circumstances under which the last session of the College was commenced, the efforts of the professors have been such as to give satisfaction to an intelligent class of students, and to inspire the friends of the institution with high hopes for the future At a meeting of the class, as the recent course was approaching its close, the following resolution was unanimously adopted:

"*Resolved*, That we tender our grateful acknowledgments to the honorable and able faculty of this institution, for the interest they have taken in exhibiting to us clearly the various subjects connected with medical science. We beg leave to express our earnest desire that their administration of its affairs may be continued so long as it may be found for their interest and pleasure."

As far as the committee are enabled to gather information from among those who have been familiar with the history of the Ohio Medical College, it is their opinion, that as respects stability and usefulness, it was never in a more flattering condition than at the present time. The Faculty are harmonious in feeling and action, and in their determination to advance the character and usefulness of the institution. It is now, as your committee believe, in a healthful and prosperous condition, and there is no reasonable ground to fear that such condition will not continue.

In conclusion, the committee would barely observe, that the Ohio Medical College not only deserves the confidence, but the protection of the Representatives of the people, and we believe the time is not far distant, when she will assume a position but little, if any, behind the best Medical institutions in the Union. The Legislature would doubtless be no less willing to favor this event than it should be our pleasure and pride to indulge in its anticipation.

Not deeming it necessary to engage in any additional legislation, the committee ask to be discharged from the further consideration of the subject."

March 8.

---

"Mr. Perkins, from the Standing Committee on Medical Colleges and Medical Societies, made the following Report:

A minority of the Standing Committee on Medical Colleges and Medical Societies, to which was referred the annual report of the trustees of the Medical College of Ohio, with instructions to inquire into the causes which retard its prosperity, have, as far as practicable in the time, and from the means within their reach, discharged the duty enjoined upon them, and beg leave to submit the following report and resolutions:

The Medical College of Ohio was chartered in the year 1819, and went into operation in the year 1820. There are no vested rights connected with it; the Board of Trustees are appointed from time to time by the General Assembly, and they are the corporators; none of its property has been derived from private individuals; the funds vested in it are those of the State, derived from auction duties in the city of Cincinnati. It was established by the Legislature, to aid the young men of Ohio in acquiring a medical education, and, in all respects, may be placed in the same class of State institutions with the school for the blind, or for the deaf and dumb. Its charter has been several times modified, at the pleasure of successive General Assemblies, and once an elaborate inquiry, by a Board of Commissioners, into its condition, was ordered and made; and finally, it is the duty of its trustees to make an annual report to the Legislature on the manner in which they have conducted the affairs of the institution, and on its actual condition.

Thus, it appears, that this corporation is in all respects a State institution, and may, at the pleasure of the General Assembly, be modified, removed, or dissolved.

By a reference to the long series of annual reports made to the General Assembly, the minority of your committee find that between 35,000 and 40,000 dollars, of the auction duties collected in Cincinnati, have been given to the College, and that this sum has been appropriated to the erection of an edifice, and the purchase of books and apparatus required in medical instruction. Thus, the State has discharged her duty towards the institution with liberality. But the same reference shows that the classes of the College, with the exception of two or three sessions, have always been small, and that the number this winter is not greater than it was ten years ago. Within the above period, the trustees have felt themselves called upon to make several re-organizations of the faculty, being embarrassed by many external and internal difficulties; and that within the last seven years, no less than fifteen different gentlemen have held professorships in it, and during the last summer, four out of six of its professors, embracing some of its oldest and ablest, without any obvious cause, resigned their places, and entered three other institutions.

One of the chairs has not yet been permanently filled, (unless done recently,) and others are filled by gentlemen residing at a distance from the institution, several of them having just entered upon their career as medical teachers, are not generally known as such, though highly respectable in private practice; hence, the attractions which the institution can exert for some years to come, must necessarily be less than it has exerted heretofore. At the same time, it has become known to your committee, that the Cincinnati College, "gotten up," as they are informed, by more than 600 contributors of that city, has established a medical, in connexion with its law and academical departments, which has a faculty of seven professors, all resident in the city, and a class of 125 pupils, or more than 50 per cent. greater than that of the Medical College of Ohio.

Under all these circumstances, the committee are of opinion that it is no longer expedient for the State to embarrass herself with the support and management of a Medical institution in that city. They believe, however, that, as moneys vested in the establishment were derived from the auction sales of the city, there would be a propriety in securing to it the advantages of its own appropriation, provided the object of education be still kept in view. Under this feeling, the committee have reflected on the new disposition which might be advantageously made of the money vested in the Medical School of the State, and have come to the following conclusion:

The Ohio Mechanics Institute, chartered several years ago, for the promotion of those mechanic arts and the branches of science connected with them, on which the prosperity of our State so vitally depends has languished for the want of a suitable edifice in which instruction could be delivered, and the products of the mechanical ingenuity of the citizens of the State, be arranged for public inspection. To this valuable school of the useful arts, the committee are of opinion, the edifice now occupied by the Medical College, should be conveyed; and with it such books and pieces of apparatus, as are especially calculated to illustrate those branches of science which it is the object of the Institote to promote. The remainder of the library, apparatus and preparations, should then, in the opinion of the committee, be turned over to the trustees of the Cincinnati College, in trust, for the benefit of its Medical Department.

By this disposition of the property of the Medical College, the State would be relieved from further responsibility, the interest of medical education would be secured, and a school for the higher mechanic arts, of incalculable value to the State, be founded and endowed in such manner as would render it comprehensive, prosperous and permanent.

In pursuance of these views, the committee ask leave to offer for adoption the following resolutions:

*Resolved by the General Assembly of the State of Ohio*, That it shall be the duty of the Trustees of the Medical College of Ohio, forthwith, to convey in fee simple to the Ohio Mechanics Institute, the public edifice of the corporation, with the grounds pertaining to the same, in the city of Cincinnati, on the condition, that it shall never, without the consent of the Legislature, be diverted from the objects for which said Institute was incorporated.

*Resolved*, That said Trustees shall deliver to the managers of said Institute, such books now in the College Library, as relates to the Science of Natural Philosophy, and the Mechanic Arts; and such portions of the apparatus of said College as may be designed to illustrate those branches of human knowledge.

*Resolved*, That all the remaining books, chemical apparatus, preparations, drawings, and other means adapted to medical inttruction in its various branches, shall by them be delivered to the Trustees of the Cincinnati College, for the use and benefit of the Medical Department of that Institution, on the condition, that should they, at any time discontinue said Department, the whole shall revert to the State.

*Resolved*, That the duties and priviliges now by law assigned to the Professors of the Medical College of Ohio, in the Commercial Hospital and Lunatic Asylum in the city of Cincinuati, shall hereafter be performed by the Medical Professors of the Cincinnati College.

*Resolved*, That the Secretary of State shall, and he is hereby directed, to transmit a certified copy of these resolutions, to the President of the Board of Trustees of the Medical College of Ohio.

